# The Perseverative Thinking Questionnaire (PTQ): Validation of a content-independent measure of repetitive negative thinking

**DOI:** 10.1016/j.jbtep.2010.12.003

**Published:** 2011-06

**Authors:** Thomas Ehring, Ulrike Zetsche, Kathrin Weidacker, Karina Wahl, Sabine Schönfeld, Anke Ehlers

**Affiliations:** a Department of Clinical Psychology, University of Amsterdam, Roetersstraat 15, 1018 WB Amsterdam, The Netherlands; b Phillips University of Marburg, Germany; c University of Lübeck, Germany; d Technical University Dresden, Germany; e King’s College London, Institute of Psychiatry, UK

**Keywords:** Rumination, Worry, Repetitive negative thinking, Questionnaire, Reliability, Validity, Transdiagnostic processes

## Abstract

Repetitive negative thinking (RNT) has been found to be involved in the maintenance of several types of emotional problems and has therefore been suggested to be a transdiagnostic process. However, existing measures of RNT typically focus on a particular disorder-specific content. In this article, the preliminary validation of a content-independent self-report questionnaire of RNT is presented. The 15-item Perseverative Thinking Questionnaire was evaluated in two studies (total *N* = 1832), comprising non-clinical as well as clinical participants. Results of confirmatory factor analyses across samples supported a second-order model with one higher-order factor representing RNT in general and three lower-order factors representing (1) the core characteristics of RNT (repetitiveness, intrusiveness, difficulties with disengagement), (2) perceived unproductiveness of RNT and (3) RNT capturing mental capacity. High internal consistencies and high re-test reliability were found for the total scale and all three subscales. The validity of the Perseverative Thinking Questionnaire was supported by substantial correlations with existing measures of RNT and associations with symptom levels and clinical diagnoses of depression and anxiety. Results suggest the usefulness of the new measure for research into RNT as a transdiagnostic process.

## Resesarch highlights

► The new questionnaire (PTQ) is a content-independent measures of repetitive negative thinking. ► The PTQ shows good reliability and validity. ► The factor structure is stable across samples. ► The PTQ enables the investigation of repetitive negative thinking across disorders.

## 1 General introduction

A number of different emotional problems have been found to be related to heightened levels of repetitive negative thinking (RNT) in the form of worry and/or rumination. For example, individuals with depressive disorders have been shown to ruminate excessively about the symptoms of depression, their causes and consequences (Nolen-Hoeksema, Wisco, & Lyubomirsky, 2008). Importantly, results from longitudinal as well as experimental studies suggest that depressive rumination is not only an epiphenomenon of the disorder, but plays a causal role in its development and maintenance (Nolen-Hoeksema et al., 2008; Watkins, 2008). Similarly, excessive worry is a key feature of generalized anxiety disorder (GAD) (APA, 2000; Borkovec, Robinson, Pruzinsky, & DePree, 1983). Although most of the research to date has focused on depression and GAD, there is evidence that heightened levels of rumination and/or worry are present in most Axis I disorder, including posttraumatic stress disorder (PTSD), social phobia, obsessive-compulsive disorder (OCD), insomnia, eating disorders, panic disorder, hypochondriasis, alcohol use disorder, psychosis and bipolar disorder (for a review see Ehring & Watkins, 2008).

Based on the widespread presence of rumination and worry across disorders, is has been suggested that RNT is a transdiagnostic process that shows the same characteristics across disorders, whereby only the content is disorder-specific (Ehring & Watkins, 2008; Harvey, Watkins, Mansell, & Shafran, 2004). Evidence supporting this view comes from four types of studies. First, self-report questionnaires measuring different types of RNT (mainly worry vs. rumination) are highly correlated and are related to symptom levels of anxiety and depression to a similar extent (e.g., Fresco, Frankel, Mennin, Turk, & Heimberg, 2002; Segerstrom, Tsao, Alden, & Craske, 2000; Siegle, Moore, & Thase, 2004). This supports the view that these questionnaires measure more or less the same process. Second, studies directly comparing characteristics of worry and depressive rumination have revealed very few differences between these processes and none of these differences has been replicated yet (Papageorgiou & Wells, 1999; Watkins, 2004; Watkins, Moulds, & Mackintosh, 2005). Third, the experimental induction of different types of RNT (typically worry vs. rumination) has been shown to lead to increased levels of anxiety and depression (e.g., Blagden & Craske, 1996; McLaughlin, Borkovec, & Sibrava, 2007). Finally, across disorders worry and rumination have been found to share a number of important characteristics; they tend to consist of thoughts rather than images, be relatively abstract and to be related to positive as well as negative meta-cognitions (for a review see Ehring & Watkins, 2008).

Taken together, these findings suggest that it may be promising to investigate RNT across disorders rather than using a disorder-focused perspective. However, research into RNT as a transdiagnostic process is complicated by the fact that current definitions and measures of this variable are mostly focused on a specific *content* and are therefore disorder-specific. For example, depressive rumination is typically defined as “repetitive and passive thinking about one’s symptoms of depression and the possible causes and consequences of these symptoms” (Nolen-Hoeksema, 2004, p. 107). Consequently, the Response Style Questionnaire (RSQ; Nolen-Hoeksema & Morrow, 1991), regarded as the standard measure of depressive rumination, focuses on depression-related repetitive thoughts. Worry in GAD has most commonly been defined as “a chain of thoughts and images, negatively affect-laden and relatively uncontrollable. The worry process represents an attempts to engage in mental problem-solving on an issue whose outcome is uncertain but contains the possibility of one or more negative outcomes” (Borkovec et al., 1983; p. 10). In line with this definition, the most commonly used measure of worry, the Penn State Worry Questionnaire (PSWQ; Meyer, Miller, Metzger, & Borkovec, 1990), focuses on the type of thoughts that are typical for GAD. Similarly, definitions and measures of rumination in PTSD are focused on repetitive thinking about the trauma and/or its consequences (Ehring, Frank, & Ehlers, 2008; Michael, Halligan, Clark, & Ehlers, 2007) and those for social phobia are centered around repetitive thoughts related to a recent social interaction (Kashdan & Roberts, 2007).

We suggest that a transdiagnostic definition of RNT would need to be focused on its characteristic process (e.g., repetitiveness, difficult to disengage from), to be independent of a specific content and to be applicable to past, present and future concerns. In addition, a definition on RNT as relevant to emotional disorders should be restricted to *dysfunctional* forms of RNT, as there is evidence that certain forms of recurrent thinking can also be beneficial (Trapnell & Campbell, 1999; Watkins, 2008). Despite considerable theoretical and empirical progress in this field (see e.g. Treynor, Gonzalez, & Nolen-Hoeksema, 2003; Watkins, 2008), there is still no consensus as to which factors distinguish between functional and dysfunctional forms of repetitive thinking. Therefore, it appears premature to include variables such as abstractness of thinking into a definition of dysfunctional RNT. However, at the very least a transdiagnostic definition of RNT should include individuals’ own perception of their thinking as being unproductive. In line with this reasoning, there is evidence that self-reported unproductiveness of repetitive thinking is associated with psychopathology over and above the pure frequency of RNT (Michael et al., 2007). In addition, as repetitive thinking captures mental capacity it has been found to be associated with self-reported as well as objective difficulties concentrating on ongoing tasks (e.g., Lyubomirsky, Kasri, & Zehm, 2003).

Based on earlier conceptualizations (see Ehring & Watkins, 2008; Segerstrom, Stanton, Alden, & Shortridge, 2003; Watkins, 2008), we suggest the following working definition: Repetitive negative thinking as relevant to emotional problems is a style of thinking about one’s problems (current, past, or future) or negative experiences (past or anticipated) that shows three key characteristics: (1a) the thinking is repetitive, (1b) it is at least partly intrusive, and (1c) it is difficult to disengage from. Two additional features of RNT are that (2) individuals perceive it as unproductive and (3) it captures mental capacity. Whereas the key characteristics represent the actual thinking *process*, the two additional features refer to individuals’ perceived *dysfunctional effects* of RNT.

Based on this working definition, the current article describeds the development and initial validation of the *Perseverative Thinking Questionnaire* (PTQ) as a content-independent measure of RNT.

## 2 Study 1

The aim of Study 1 was to investigate the factor structure, reliability and validity of the German version of the new questionnaire measure in three samples.

### 2.1 Method

#### 2.1.1 Participants

*Sample 1: Internet sample*. The first sample consisted of volunteers who filled in the questionnaires via a web-based, secured and encrypted survey. All participants with complete data on the Perseverative Thinking Questionnaire were included in the analyses (*N* = 724; age: *M* = 30.05, *SD* = 10.58; 73% female). Participants for this sample were recruited by posting information about the study and a link to the online questionnaire on a number of websites advertising web-based studies.

*Sample 2: Non-clinical sample*. The second sample consisted of *N* = 501 non-clinical participants (age: *M* = 26.59, SD = 7.89; 77% female). Seventy-nine percent of participants in this sample were University students, 21% of participants were recruited from the general population. Participants in this sample filled in pencil-and-paper versions of all questionnaires.

*Sample 3: Clinical sample*. The third sample consisted of *N* = 113 clinical participants (age: *M* = 43.22, *SD* = 11.35; 52% female). Participants were recruited from the patient population of two mental health clinics. The primary diagnoses in this sample were major depressive disorder (39.8%), an anxiety disorder (24.8%), or other disorders (adjustment disorder: 13.3%; somatoform disorder: 10.6%; substance use disorder: 8.8%; bulimia nervosa: 2.7%). These diagnoses were clinical diagnoses established during the pre-treatment assessment.

#### 2.1.2 Measures

For all questionnaires, German-language versions were used.

##### 2.1.2.1 Perseverative Thinking Questionnaire (PTQ)

Based on the working definition of repetitive negative thinking described in the introduction and some pilot data (Zetsche, Ehring, & Ehlers, 2009), the PTQ was developed, consisting of 15 items. The item pool comprised three items for each of the assumed process characteristics of repetitive negative thinking: (1a) repetitive (e.g., “The same thoughts keep going through my mind again and again”), (1b) intrusive (e.g., “Thoughts come to my mind without me wanting them to”), (1c) difficult to disengage from (e.g., “I can’t stop dwelling on them”), (2) unproductive (e.g., “I keep asking myself questions without finding an answer”), (3) capturing mental capacity (e.g. “My thought prevent me from focusing on other things”) (see Appendix for all 15 items). Participants were asked to rate each item on a scale ranging from ‘0’ (*never*) to ‘4’ (*almost always*).

##### 2.1.2.2 Other measures of RNT

In order to establish convergent validity, a number of existing measures of RNT were used.

The rumination scale of the *Response Style Questionnaire* (*RSQ*; Nolen-Hoeksema, 1991; German version: Kühner, Huffziger, & Nolen-Hoeksema, 2007) was used to assess repetitive negative thinking in the form of depressive rumination. The questionnaire consists of 22 items describing the individual’s response to sad or depressed mood (e.g., “Think about how passive and unmotivated you feel”) that are rated on a scale from ‘1’ (*never*) to ‘4’ (*always*). The RSQ is regarded as the standard measure of rumination; it has been used widely in clinical as well as non-clinical populations and has demonstrated high reliability and validity (Kühner et al., 2007; Luminet, 2004; Nolen-Hoeksema, 2004). A total score of the 22 items was computed as well as the two subscales *brooding* (5 items) and *reflection* (5 items). Earlier research has shown that brooding in particular represents the dysfunctional style of depressive rumination that is related to current and future symptoms of depression (Treynor et al., 2003).

The *Penn State Worry Questionnaire* (PSWQ) (Meyer et al., 1990; German version: Stöber, 1998) was used to assess repetitive negative thinking in the form of worry. It consists of 16 items (e.g., “Many situations make me worry”) that are rated on a scale from ‘1’ (*not at all typical*) to ‘5′ (*very typical*). The PSWQ has been shown to possess good psychometric properties in non-clinical and clinical samples. (Meyer et al., 1990; Stöber, 1998).

A subgroup of non-clinical participants (*n* = 219) also filled in the *Rumination Scale* (McIntosh, Harlow, & Martin, 1995). This self-report questionnaire is based on Martin and Tesser’s (1996) goal-discrepancy theory of rumination and attempts to assess the frequency of rumination in people’s daily lives. There is preliminary data supporting the reliability and validity of the measure (McIntosh et al., 1995).

##### 2.1.2.3 Depression and anxiety

Symptom levels of depression were assessed with the Beck Depression Inventory (BDI; Beck, Rush, Shaw, & Emery, 1979; German version: Hautzinger, Bailer, Worall, & Keller, 1995). The trait version of the State Trait Anxiety Inventory (STAI; Spielberger, Gorsuch, Lushene, Vagg, & Jacobs, 1983; German version: Laux, Glanzmann, Schaffner, & Spielberger, 1981) was used to assess levels of anxiety. Both questionnaires are widely used and have been shown to possess good psychometric properties in non-clinical and clinical samples (Beck, Steer, & Garbin, 1988; Spielberger et al., 1983).

#### 2.2 Results

##### 2.2.1 Confirmatory factor analyses

**Fig. 1:**
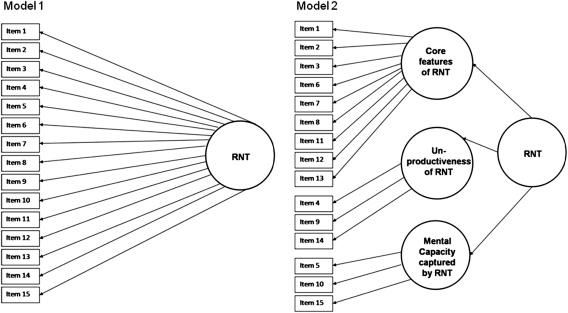
Schematical Representation of the Two Models Tested in the CFAs. *Note*. Model 1 = Single common factor model; Model 2 = Second-order single-factor model with three lower-order factors.

A confirmatory factor analysis using LISREL 8.54 was conducted to test the goodness-of-fit for two different models (see Fig. 1). Model 1 represents a single common factor model in which all 15 items load on one underlying factor reflecting RNT. Model 2 is based on the working definition underlying the PTQ and includes one higher-order factor (RNT) and three lower-order factors, namely *Core Characteristics of RNT*, *Unproductiveness of RNT* and *RNT Capturing Mental Capacity*.

First, separate CFAs were conducted in each sample. The data expressed multivariate non-normality in the three samples, (Sample 1: Mardia’s test of multivariate kurtosis = 28.83, *p* < .001; Small’s test of multivariate normality = 280.78, *p* < .001; Sample 2: Mardia’*s* = 15.16, *p* < .001; Small’*s* = 167.89, *p* < .001; Sample 3: Mardia’*s* = 7.05, *p* < .001; Small’*s* = 90.07, *p* < .001). Due to the multivariate non-normality of the data and the ordinal nature of the items, the fit of the individual models was investigated with robust maximum likelihood estimation based on the polychoric correlation matrix and the asymptotic covariance matrix, calculated from equal thresholds for multi-group confirmatory factor analyses. Following this procedure, model fit indices corrected for non-normality were obtained, such as the robust Satorra–Bentler scaled test statistic (Hu, Bentler, & Kano, 1992). Since the chi-square statistic increases with sample size, leading to rejection of the hypothesized model even at good fit (Bentler & Bonett, 1980), additional fit indices were also examined, namely the Root Mean Square Error of Approximation (RMSEA; acceptable fit: .05–.08, good fit: 0–.05), the Standardized Root Mean Square Residual (SRMR; acceptable fit: .05–.10, good fit: 0–.05), the Comparative Fit Index (CFI; acceptable fit: .95–.97, good fit: .97–1) and the Consistent version of the Akaike Information Criterion (CAIC) (see Hu & Bentler, 1998; Schermelleh-Engel, Moosbrugger, & Müller, 2003). Fit indices of the confirmatory factor analyses per sample are shown in Table 1. Model 2 (one higher-order factor and three lower-order factors) fit best with the data, showed an acceptable to good overall model fit across groups and fit indices and was identified as best fitting model according to significant ∆CAIC in all three groups. The fit for Model 1 was inadequate, indicated by higher RMSEA, SRMR and lower CFI values. According to the completely standardized solution of Model 2, the factor loading of the items were between .61 and .93 and the loadings of the subfactors on the superordinate factor were .90–.98 for Factor 1, .92–.97 for Factor 2 and .79–.83 for Factor 3. The three lower-order factors were intercorrelated with *r* = .85–.94 (Factors 1 and 2), *r* = .74–.79 (Factors 1 and 3) and *r* = .72–.78 (Factors 2 and 3).

In a second step, sequential multi-group confirmatory factor analyses were conducted to assess configural invariance, metric invariance and invariance of the error variances across groups (Byrne, Shavelson, & Muthén, 1989; Hoyle & Smith, 1994). The second-order model with three lower-order factors (Model 2) identified as the best fitting model in all three single-group CFAs described above was used as the baseline model. The baseline model fits the same pattern of fixed and non-fixed model parameters in all three groups simultaneously and imposes no additional equality constraints across groups. Table 2 summarizes the global fit indices for the performed steps in invariance testing. The baseline model of configural invariance expressed acceptable to good fit and was compared to a more restricted model in which all first-order factor loadings were set equal across groups to assess metric invariance. Significant differences between the Satorra–Bentler scaled chi-square statistic of the baseline model and a more constrained model indicated that full metric invariance was untenable. However, partial metric invariance across samples was established when allowing the first-order loading of item 3 and item 13 to vary between groups while holding all remaining first-order loadings invariant. The partial metric invariant model served as the new baseline model for testing invariance of error variances across groups. While full invariance of error variances was untenable, the hypothesis of partial invariance of error variances of the PTQ was confirmed when allowing the error variance of item 15 to vary between groups. The standardized factor loadings on the lower-order factors are shown in Table 3 for the final partially invariant model across samples included in Study 1.

##### 2.2.2 Reliability

###### 2.2.2.1 Internal consistency

For the total sum score, an excellent internal consistency was found in all three samples (Sample 1: *α* = .95; Sample 2: *α* = .94; Sample 3: *α* = .95). Similarly, all subscales showed good internal consistencies (*Core Characteristics of RNT*: *α* = .92–.94; *Unproductiveness of RNT*: *α* = .77–.87; *RNT Capturing Mental Capacity*: .82–.90).

###### 2.2.2.2 Re-test reliability

In order to establish re-test reliability, a subgroup of *n* = 186 participants filled in the PTQ twice with a 4 week interval. Results showed a satisfactory test–retest correlation for the PTQ total score (*r*_tt_ = .69; *p* < .001) as well as the three subscores: *Core Characteristics of RNT* (*r*_tt_ = .66; *p* < .001), *Unproductiveness of RNT* (*r*_tt_ = .68; *p* < .001), *RNT Capturing Mental Capacity* (*r*_tt_ = .69; *p* < .001).

##### 2.2.3 Convergent validity

The total PTQ score showed significant and substantial correlations with other measures of RNT, namely the RSQ (*r* = .72), the PSWQ (*r* = .70) and the Rumination Scale (*r* = .62). Similarly, all PTQ subscales showed significant and substantial correlations with these measures (see Table 4). In a series of multilevel regression analyses, we tested whether the associations of the PTQ with other measures of RNT significantly differed between the three subsamples. No significant variance in intercepts or slopes was found across groups (all *p* > .34). This shows that the PTQ was similarly related to the other RNT measures in all three subsamples.

For the RSQ subscales, the PTQ total scale showed a significantly higher correlation with the RSQ brooding subscale (*r* = .63) than with the RSQ reflection subscale (*r* = .42), *t*(1254) = 8.66, *p* < .001. Similarly, the significantly higher correlations with brooding compared to those with reflection were found for all the PTQ subscale scores, *Core Characteristics of RNT*, *t*(1254) = 7.14, *p* < .001, *Unproductiveness of RNT, t*(1254) = 11.05, *p* < .001, and *RNT Capturing Mental Capacity*, *t*(1254) = 4.98, *p* < .001.

##### 2.2.4 Predictive validity

The PTQ total score was significantly and substantially associated with symptom levels of depression as assessed with the BDI (*r* = .54). This correlation did not differ significantly from correlations of the BDI with established measures of RNT, namely the RSQ, *r* = .53, *t*(600) = .31, *p* = .76, and the PSWQ, *r* = .55, *t*(355) = .27, *p* = .79. However, it was significantly higher than the correlation between the BDI and the Rumination Scale, *r* = .38, *t*(216) = 2.72, *p* < .01.

Similarly, the PTQ was significantly correlated with symptom levels of anxiety, assessed with the STAI (*r* = .64). This correlation did not differ significantly from the correlation of the STAI with the PSWQ, *r* = .70, *t*(211) = 1.30, *p* = .19. However, it was significantly higher than the association of the STAI with the RSQ, *r* = .50, *t*(211) = 2.89, *p* < .01, and the Rumination Scale, *r* = .53, *t*(211) = 2.02, *p* < .05.

Significant and substantial correlations with symptom levels of anxiety and depression were also found for all PTQ subscale scores (see Table 4). In multilevel regression analyses, no significant variance in intercepts or slopes was found across groups, showing that the association between PTQ scores and symptom levels of anxiety and depression were similar in all subgroups.

In order to test whether PTQ scores differed between diagnostic groups, an ANOVA was conducted with the PTQ sum score as the dependent variable and diagnostic group (4 groups: no disorder, depression, anxiety disorder, other disorder) as the independent variable. The no disorder group in this analysis comprised a random sample of *n* = 100 participants from Samples 1 and 2 that was matched by age and gender to the clinical groups. A significant main effect of diagnostic group was found, *F*(3, 209) = 7.69, *p* < .001, *η*_p_^2^ = .10. Planned simple contrasts between each of the clinical groups and participants without psychological disorder showed that participants without a current disorder had significantly lower PTQ scores (*M* = 28.14, *SD* = 13.23) than patients suffering from depression (*M* = 37.56, *SD* = 9.99; *p* < .001) or anxiety disorders (*M* = 35.93, *SD* = 13.60; *p* < .001). However, patients suffering from other disorders (*M* = 28.60, *SD* = 12.77) did not significantly differ from the no disorder group (*p* = .85). The same pattern of results was found for the three PTQ subscales *Core Characteristics of RNT*, *F*(3, 209) = 6.49, *p* < .001, *η*_p_^2^ = .09, *Unproductiveness of RNT, F*(3, 209) = 6.89, *p* < .001, *η*_p_^2^ = .09, and *RNT Capturing Mental Capacity, F*(3, 209) = 6.50, *p* < .001, *η*_p_^2^ = .09.

#### 2.3 Discussion

The results of this study are promising. Results of the CFA clearly supported the assumed factor structure of the measure, with one higher-order factor representing RNT in general as well as three lower-order factors representing the *Core Characteristics of RNT (Repetitiveness, Intrusiveness, Difficulties to Disengage)*, *Unproductiveness of RNT* and the degree to which RNT captures *Mental Capacity*. Importantly, this factor structure was replicated in all three subsamples and results from multi-group CFAs showed invariance of the model across samples for the vast majority of the PTQ items. All PTQ scales showed high internal consistencies and a satisfactory stability.

The validity of the measure was tested in two ways. First, correlations between the PTQ and established measures of RNT were computed. Substantial correlations with all measures were found and these associations were similar across samples. Interestingly, the PTQ scores correlated significantly higher with the *RSQ* subscale brooding than the reflection subscale of the same instrument. In earlier research, the brooding subscale has been found to represent the more dysfunctional form of rumination that is related to the onset and maintenance of symptoms of depression, whereas reflection may be a more adaptive form of recurrent thinking as it has been found to be related to less future symptoms of depression (see Treynor et al., 2003). The finding therefore supports the view that the *PTQ* measures a dysfunctional form of RNT. This idea is also in line with the results regarding the predictive validity of the *PTQ*. The new questionnaire was found to be significantly and substantially related to levels of depression and anxiety. Importantly, the PTQ was found to predict these symptom measures as well as the standard measures of dysfunctional RNT (the RSQ and the PSWQ). The validity of the questionnaire was further supported by the finding that participants currently suffering from depression or anxiety disorders scored significantly higher on the questionnaire than those without disorders.

## 3 Study 2

The aim of Study 2 was to replicate the findings of Study 1 for the English version of the PTQ.

### 3.1 Method

#### 3.1.1 Participants

The sample for Study 2 comprised 494 participants (age: *M* = 28.20, *SD* = 12.43; 71.5% female), who had filled in the English-language version of the *Perseverative Thinking Questionnaire* by means of a web-based, secure and encrypted survey. Participants were recruited through websites advertising web-based studies and among students of the University of Miami.

#### 3.1.2 Materials

##### 3.1.2.1 Perseverative Thinking Questionnaire

The PTQ was translated into English and backtranslated in order to establish equivalence of the two language versions. The English PTQ was used in this study.

##### 3.1.2.2 Other measures of RNT

As in Study 1, the Penn State Worry Questionnaire (PSWQ; Meyer et al., 1990) and the Response Style Questionnaire (RSQ; Nolen-Hoeksema & Morrow, 1991) were used as established measures of RNT.

##### 3.1.2.3 Depression

The Inventory of Depressive Symptomatology (IDS; Rush, Gullion, Basco, Jarrett, & Trivedi, 1996) was used to assess symptoms of depression. The IDS has been shown to be a reliable and valid self-report instrument of current symptom levels of depression (Rush et al., 1996).

#### 3.2 Results

##### 3.2.1 Confirmatory factor analysis

As for Study 1, single-group CFA in LISREL 8.54 was used to test the goodness-of-fit for the two different models (see Fig. 1). The analysis was based on the polychoric correlation matrix and the asymptotic covariance matrix due to non-normal distribution of the ordinal PTQ items (Mardia’s test of multivariate kurtosis = 32.52, *p* < .001; Small’s test of multivariate normality = 221.18, *p* < .001). As in Study 1, the fit indices suggested best performance for Model 2 (see Table 1), supported by a significant ∆CAIC. RMSEA for Model 2 was in the acceptable range, and the fit indices SRMR and CFI indicated a good fit of the model, whereas Model 1 showed inadequate fit.

The standardized factor loading for the PTQ items on the three lower-order factors are shown in Table 3. The standardized factor loadings for the three lower-order factors on the superordinate factor were .90 for *Factor 1*, .94 for *Factor 2,* and .90 for *Factor 3*. Intercorrelations between the three lower-order factors were *r* = .85 (Factors 1 and 2; Factors 2 and 3) and *r* = .81 (Factors 1 and 3).

##### 3.2.2 Internal consistency

Excellent internal consistencies were found for the total scale (*α* = .95) as well as all three subscales (*Factor 1*: *α* = .94; *Factor 2: α* = .83; *Factors 3*: *α* = .86).

##### 3.2.3 Convergent validity

All PTQ scales showed significant and substantial correlations with the RSQ and the PSWQ as established measures of RNT (see Table 4). The correlation with the RSQ brooding subscale was significantly higher than the correlation with the RSQ reflection subscale for the PTQ total scale, *t*(457) = 2.61, *p* < .01, as well the subscales *Core Characteristics of RNT, t*(457) = 2.31, *p* < .05, and *Perceived Unproductiveness*, *t*(457) = 3.54, *p* < .001, but equally high for *RNT Capturing Mental Capacity*, *t*(457) = 1.07, *p* = .28.

##### 3.2.4 Predictive validity

All *PTQ* scores were significantly and substantially correlated with the *IDS* measuring current symptom levels of depression (see Table 4). The correlation of symptom levels of depression with the PTQ total score (*r* = .58) did not significantly differ from the correlations of the IDS with the RSQ (*r* = .63), *t*(457) = 1.31, *p* = .19, and the PSWQ (*r* = .58), *t*(457) = 0, *p* = 1.

#### 3.3 Discussion

The study replicated the findings of Study 1. Confirmatory factor analyses showed that the English version of the *PTQ* shows the same factor structure as the German version with one higher-level factor representing RNT in general as well as three lower-order factors. The total scale and the subscales were found to be highly internally consistent. In addition, significant correlations with established measures of RNT and levels of depression support the validity of the measure.

## 4 General discussion

The aim of the studies described in this article was to provide a preliminary test of a content-independent measure of repetitive negative thinking. The 15-item *Perseverative Thinking Questionnaire* was developed based on a working definition of RNT that includes three core characteristics of RNT (repetitiveness, intrusiveness and difficulties to disengage) as well as two associated features (unproductiveness, capturing mental capacity). The factor structure and psychometric properties were investigated in two studies (total *N* = 1832). Taken together, the results provide preliminary evidence for the usefulness, reliability and validity of the *PTQ* as measure of RNT.

In both studies, two different models were compared using confirmatory factor analyses. A second-order model with one higher-order factor and three lower-order factors provided a good fit with the data and performed significantly better than a single common factor model. This factor structure was found to be very robust as it provided a good fit with the data in non-clinical as well as clinical participants and for both English and German versions of the RNT. Results of multi-group CFA support partial invariance of the model parameters across samples. Importantly, this model is in line with the theoretical model and the working definition underlying the PTQ. The higher-order factor represents the concept of RNT as a whole, whereas the first lower-order factor represents the core characteristics of RNT (repetitiveness, intrusiveness and difficulties to disengage) and the other lower-order factors represent the additional features of unproductiveness (Factor 2) and mental capacity captured by RNT (Factor 3). Reassuringly, all items loaded highly on the factor they had a priori been assigned to. Based on the results of the CFA across studies, we recommend computing a total PTQ score as the sum of all 15 items. In addition, three subscale scores can be computed.

Across studies and samples, the PTQ total scale as well as the subscales showed excellent internal consistencies. In addition, results from Study 1 suggest that the measure shows adequate re-test reliability, further supporting the reliability of the measure.

Both studies tested the concurrent validity of the PTQ by correlating its scores with those of established measures of RNT, most important the PSWQ as the standard measure of excessive worry and the RSQ as the standard measure of depressive rumination. Across studies and samples, substantial correlations were found. Therefore, the PTQ can be regarded as a valid measure of RNT. These findings also suggest that it is possible to assess the process characteristics of RNT independent of its content. Existing measures of RNT have been criticized as being highly content-dependent (see Ehring & Watkins, 2008; Luminet, 2004; Treynor et al., 2003). The new measure avoids these problems by focusing on the characteristic process of RNT.

There is accumulating evidence that not all forms of recurrent thinking are dysfunctional (see Trapnell & Campbell, 1999; Watkins, 2008). The PTQ was developed with the aim to assess dysfunctional forms of RNT that are involved in the maintenance of emotional disorders. The validity of the PTQ as a measure of dysfunctional RNT was investigated in two ways. First, we looked at associations of the PTQ with the two subscales of the RSQ. In line with the hypotheses, the PTQ was found to be significantly more strongly related to the brooding subscale of the RSQ, representing dysfunctional rumination, than to the reflection subscale of this measure, which has been found to be related to less symptomatology in the long run (Treynor et al., 2003). Secondly, the PTQ was found to be significantly associated with symptom levels of depression and anxiety as well as current diagnoses of depression and anxiety disorders. Importantly, the associations of these symptom levels with the PTQ were as high as those with the RSQ and the PSWQ and significantly higher than those with the Rumination Scale. Interestingly, all PTQ subscales appear to be representing dysfunctional RNT to a similar degree as all three subscales were equally related to symptom severity measures. As a whole, the results support the validity of the PTQ as a measure of dysfunctional RNT as relevant to emotional disorders.

A number of limitations of the current studies are noteworthy. First, the subscales *Unproductiveness* and *RNT Capturing Mental Capacity* only comprise three items each. Although these scales showed acceptable internal consistencies in all samples, it may be advisable to extend these scales in order to increase their reliability. Second, no structured clinical interviews were used to establish clinical diagnoses and no information regarding secondary diagnoses was available. Future studies should use carefully diagnosed groups to further investigate the transdiagnostic properties of the questionnaire. Third, the study mainly focused on the relationship between the PTQ with symptom levels of depression and anxiety. However, RNT has been found in earlier research to be related to other types of emotional problems, too (see Ehring & Watkins, 2008). More research is needed to investigate the relationship between PTQ scores and a wider range of psychopathology. Unexpectedly, participants suffering from emotional disorders other than anxiety disorders or depression did not differ significantly from the non-clinical group in Study 1. At first sight, this is surprising as earlier research has found elevated levels of RNT in almost all axis-I disorders (Ehring & Watkins, 2008). It is noteworthy, however, that the majority of participants in this group had received a prior diagnosis of either an adjustment disorder or a somatoform disorder. To our knowledge, there are no published studies showing an association between RNT and these disorders to date. Future research including a wider range of emotional disorders is needed to test whether PTQ scores are related to disorders other than depression and anxiety disorders. Finally, the present studies exclusively focused on the association between the PTQ and other self-report measures. Future studies should test whether the PTQ can also predict behavioral measures of RNT, such as the number of steps in the catastrophizing interview (Vasey & Borkovec, 1992).

Despite these limitations, the current studies provide important preliminary evidence for the *Perseverative Thinking Questionnaire* as a useful, reliable and valid measure of repetitive negative thinking that may help to facilitate transdiagnostic research into this cognitive process.

## Figures and Tables

**Table:** Instruction: In this questionnaire, you will be asked to describe how you *typically* think about negative experiences or problems. Please read the following statements and rate the extent to which they apply to you when you think about negative experiences or problems.

		never	rarely	sometimes	often	almost always
1.	The same thoughts keep going through my mind again and again.	0	1	2	3	4
2.	Thoughts intrude into my mind.	0	1	2	3	4
3.	I can’t stop dwelling on them.	0	1	2	3	4
4.	I think about many problems without solving any of them.	0	1	2	3	4
5.	I can’t do anything else while thinking about my problems.	0	1	2	3	4
6.	My thoughts repeat themselves.	0	1	2	3	4
7.	Thoughts come to my mind without me wanting them to.	0	1	2	3	4
8.	I get stuck on certain issues and can’t move on.	0	1	2	3	4
9.	I keep asking myself questions without finding an answer.	0	1	2	3	4
10.	My thoughts prevent me from focusing on other things.	0	1	2	3	4
11.	I keep thinking about the same issue all the time.	0	1	2	3	4
12.	Thoughts just pop into my mind.	0	1	2	3	4
13.	I feel driven to continue dwelling on the same issue.	0	1	2	3	4
14.	My thoughts are not much help to me.	0	1	2	3	4
15.	My thoughts take up all my attention.	0	1	2	3	4

**Table 1:** Results of Group-wise Confirmatory Factor Analyses.

	S-B χ^2^	df	RMSEA [90% CI]	SRMR	CFI	CAIC	∆CAIC
***Study 1***							
*Sample 1 (Internet)*							
Model 1	1195.09^∗∗∗^	90	.13 [.12, .14]	.068	.93	1422.64	762.45
Model 2	409.90^∗∗∗^	87	.072 [.065, .079]	.038	.97	660.19	.00
*Sample 2 (Non-clinical)*							
Model 1	463.34^∗∗∗^	90	.092 [.084, .10]	.058	.96	679.42	194.82
Model 2	246.91^∗∗∗^	87	.061 [.052, .070]	.039	.98	484.60	.00
*Sample 3 (Clinical)*							
Model 1	176.84^∗∗∗^	90	.094 [.073,.11]	.065	.92	347.86	33.55
Model 2	126.20^∗∗^	87	.064 [.037, .088]	.057	.95	314.31	.00
***Study 2***							
Model 1	669.92^∗∗∗^	90	.11 [.11, .12]	.060	.94	886.00	311.30
Model 2	337.02^∗∗∗^	87	.076 [.068, .085]	.045	.97	574.70	.00

*Note*: Model 1 = Single common factor model; Model 2 = Second-order single-factor model, with three lower-order factors; S-B χ^2^ = Satorra–Bentler scaled chi-square statistic; *df* = degrees of freedom; RMSEA = Root Mean Square Error of Approximation; SRMR = Standardized Root Mean Square Residual; CFI = Comparative Fit Index; CAIC = Consistent version of the Akaike Information Criterion; ∆CAIC = obtained difference between CAIC values of the tested models, lowest CAIC set to zero. ^∗∗∗^*p* < .001, ∗∗*p* < .01.

**Table 2:** Results of Multi-group Confirmatory Factor Analyses testing for Invariance of the PTQ in Study 1.

Model		S-B χ^2^	df	RMSEA [90% CI]	CFI	Model Comparison	∆ *df*	∆ S-B χ^2^
1	Baseline Model = Configural Invariance	868.83	261	.073 [.067, .078]	.97	–	–	–
2	Invariance of all first-order loadings = Full Metric Invariance	941.01	285	.072 [.067, .077]	.97	2 vs. 1	24	46.40∗
3	Invariance of all first-order loadings, freeing items 3 and 13 = Partial Metric Invariance	924.60	281	.072 [.067, .077]	.97	3 vs. 1	20	27.30
4	Model 3 + full invariance of error variances	895.92	311	.065 [.060, .070]	.97	4 vs. 3	30	50.53∗
5	Model 3 + partial invariance of error variances, freeing the error of item 15	870.20	309	.064 [.059, .069 ]	.97	5 vs. 3	28	37.34

*Note*: The baseline model refers to the simultaneous testing of one higher-order factor and three lower-order factors in all three groups. S-B χ^2^ = Satorra–Bentler scaled chi-square statistic; *df* = degrees of freedom; RMSEA = Root Mean Square Error of Approximation; CFI = Comparative Fit Index; ∆S-B χ^2^ = obtained difference between scaled S-B χ^2^ statistics of the tested models, according to Satorra and Bentler (2001); ∆df = difference in degrees of freedom between the compared models. ∗*p* < .05.

**Table 3:** Standardized Factor Loadings for Model 2.

Item	Study 1									Study 2		
	Sample 1: Internet			Sample 2: Non-clinical			Sample 3: Clinical					
	F 1	F 2	F 3	F 1	F 2	F 3	F 1	F 2	F 3	F 1	F 2	F 3
1	.83∗			.83∗			.83∗			.83∗		
2	.82∗			.82∗			.82∗			.82∗		
3	.83∗			.88∗			.81∗			.85∗		
4		.78∗			.78∗			.78∗			.84∗	
5			.83 ∗			.83∗			.83∗			.85∗
6	.85∗			.85∗			.85∗			.88∗		
7	.81∗			.81∗			.81∗			.81∗		
8	.85∗			.85∗			.85∗			.80∗		
9		.86 ∗			.86∗			.86∗			.88∗	
10			.90∗			.90∗			.90∗			.90∗
11	.83∗			.83∗			.83∗			.87∗		
12	.64∗			.64∗			.64∗			.81∗		
13	.84∗			.81∗			.77∗			.84∗		
14		.81∗			.81∗			.81∗			.81∗	
15			.88∗			.88∗			.88∗			.86∗

*Note*: F1 = Factor 1 (Core Characteristics of RNT); F2 = Factor 2 (Unproductiveness of RNT); F3 = Factor 3 (RNT capturing mental capacity); First-order loadings of Study 1 reflect the common metric completely standardized solution with partial metric invariance, allowing items 3 and 13 to vary, and partial invariance of error variances, allowing the error variance of item 15 to vary between groups. First-order loadings of Study 2 reflect the completely standardized solution ∗*p* < .05.

**Table 4:** Association of the PTQ with other Measures of Repetitive Negative Thinking, Depression, and Anxiety.

	Perseverative Thinking Questionnaire (PTQ)			
	Total Scale	Factor 1	Factor 2	Factor 3
		Core Characteristics	Unproductiveness	Capturing Mental Capacity
***Study 1***				
*Measures of Repetitive Negative Thinking*				
RSQ – total scale^a^	.72^∗∗∗^	.67^∗∗∗^	.66^∗∗∗^	.62^∗∗∗^
RSQ – brooding^a^	.63^∗∗∗^	.60^∗∗∗^	.61^∗∗∗^	.49^∗∗∗^
RSQ – reflection^a^	.42^∗∗∗^	.42^∗∗∗^	.34^∗∗∗^	.35^∗∗∗^
PSWQ^b^	.70^∗∗∗^	.68^∗∗∗^	.65^∗∗∗^	.54^∗∗∗^
Rumination Scale^c^	.62^∗∗∗^	.58^∗∗∗^	.51^∗∗∗^	.56^∗∗∗^
*Symptom levels*				
Depression (BDI)^d^	.54^∗∗∗^	.49^∗∗∗^	.54^∗∗∗^	.46^∗∗∗^
Anxiety (STAI)^e^	.64^∗∗∗^	.60^∗∗∗^	.59^∗∗∗^	.50^∗∗∗^
***Study 2***				
*Measures of Repetitive Negative Thinking*				
RSQ – total scale^f^	.59^∗∗∗^	.56^∗∗∗^	.55^∗∗∗^	.49^∗∗∗^
RSQ – brooding^f^	.54^∗∗∗^	.52^∗∗∗^	.52^∗∗∗^	.41^∗∗∗^
RSQ – reflection^f^	.43^∗∗∗^	.41^∗∗∗^	.37^∗∗∗^	.36^∗∗∗^
PSWQ^g^	.48^∗∗∗^	.46^∗∗∗^	.45^∗∗∗^	.37^∗∗∗^
*Symptom levels*				
Depression (IDS)^h^	.58^∗∗∗^	.53^∗∗∗^	.52^∗∗∗^	.52^∗∗∗^

^∗∗^*p* < .01. ^∗∗∗^*p* < .001. RSQ = Response Style Questionnaire PSWQ = Penn State Worry Questionnaire BDI = Beck Depression Inventory STAI = State Trait Anxiety Inventory IDS = Inventory of Depressive Symptomatology.

a *n* = 1258.

b *n* = 1036.

c *n* = 219.

d *n* = 603.

e *n* = 214.

f *n* = 460.

g *n* = 443.

h *n* = 343.

## References

[bib1] APA (2000). Diagnostic and Statistical Manual of mental disorders, Fourth Edition - Text Revision (DSM-IV-TR).

[bib2] Beck A.T., Rush A.J., Shaw B.F., Emery G. (1979). Cognitive therapy of depression.

[bib3] Beck A.T., Steer R.A., Garbin M.G. (1988). Psychometric properties of the Beck Depression Inventory: Twenty-five years of evaluation. Clinical Psychology Review.

[bib4] Bentler P.M., Bonett D.G. (1980). Significance tests and goodness of fit in the analysis of covariance structures. Psychological Bulletin.

[bib5] Blagden J.C., Craske M.G. (1996). Effects of active and passive rumination and distraction: a pilot replication with anxious mood. Journal of Anxiety Disorders.

[bib6] Borkovec T.D., Robinson E., Pruzinsky T., DePree J.A. (1983). Preliminary exploration of worry: some characteristics and processes. Behaviour Research and Therapy.

[bib7] Byrne B.M., Shavelson R.J., Muthén B. (1989). Testing for the equivalence of factor covariance and mean structures: the issue of partial measurement invariance. Psychological Bulletin.

[bib8] Ehring T., Frank S., Ehlers A. (2008). The role of rumination and reduced concreteness in the maintenance of posttraumatic stress disorder and depression following trauma. Cognitive Therapy and Research.

[bib9] Ehring T., Watkins E.R. (2008). Repetitive negative thinking as a transdiagnostic process. International Journal of Cognitive Psychotherapy.

[bib10] Fresco D.M., Frankel A.N., Mennin D.S., Turk C.L., Heimberg R.G. (2002). Distinct and overlapping features of rumination and worry: the relationship of cognitive production to negative affective states. Cognitive Therapy and Research.

[bib11] Harvey A.G., Watkins E., Mansell W., Shafran R. (2004). Cognitive behavioural processes across psychological disorders.

[bib12] Hautzinger M., Bailer M., Worall H., Keller F. (1995). Beck-Depressions-Inventar (BDI). Testhandbuch.

[bib13] Hoyle R.H., Smith G.T. (1994). Formulating clinical research hypotheses as structural equation models: a conceptual overview. Journal of Consulting and Clinical Psychology.

[bib14] Hu L.-T., Bentler P.M. (1998). Fit indices in covariance structure modeling: Sensitivity to underparameterized model misspecification. Psychological Methods.

[bib15] Hu L.-T., Bentler P.M., Kano Y. (1992). Can test statistics in covariance structure analysis be trusted?. Psychological Bulletin.

[bib16] Kashdan T.B., Roberts J.E. (2007). Social anxiety, depressive symptoms, and post-event rumination: affective consequences and social contextual influences. Journal of Anxiety Disorders.

[bib17] Kühner C., Huffziger S., Nolen-Hoeksema S. (2007). Response Styles Questionnaire: German version.

[bib18] Laux L., Glanzmann P., Schaffner P., Spielberger C.D. (1981). Das State-Trait-Angstinventar (STAI).

[bib19] Luminet O., Papageorgiou C., Wells A. (2004). Measurement of depressive rumination and associated constructs. Depressive rumination: Nature, theory and treatment.

[bib20] Lyubomirsky S., Kasri F., Zehm K. (2003). Dysphoric rumination impairs concentration on academic tasks. Cognitive Therapy and Research.

[bib21] Martin L.L., Tesser A., Wyer R.S. (1996). Some ruminative thoughts. Ruminative thoughts.

[bib22] McIntosh W.D., Harlow T.F., Martin L.L. (1995). Linkers and nonlinkers: goal beliefs as a moderator of the effects of everyday hassles on rumination, depression, and physical complaints. Journal of Applied Social Psychology.

[bib23] McLaughlin K.A., Borkovec T.D., Sibrava N.J. (2007). The effects of worry and rumination on affect states and cognitive activity. Behavior Therapy.

[bib24] Meyer T.J., Miller M.L., Metzger R.L., Borkovec T.D. (1990). Development and validation of the Penn State Worry Questionnaire. Behaviour Research and Therapy.

[bib25] Michael T., Halligan S.L., Clark D.M., Ehlers A. (2007). Rumination in posttraumatic stress disorder. Depression and Anxiety.

[bib26] Nolen-Hoeksema S. (1991). Responses to depression and their effects on the duration of depressive episodes. Journal of Abnormal Psychology.

[bib27] Nolen-Hoeksema S., Papageorgiou C., Wells A. (2004). The response styles theory. Depressive rumination: Nature, theory and treatment.

[bib28] Nolen-Hoeksema S., Morrow J. (1991). A prospective study of depression and posttraumatic stress symptoms after a natural disaster: the 1989 Loma Prieta earthquake. Journal of Personality and Social Psychology.

[bib29] Nolen-Hoeksema S., Wisco B.E., Lyubomirsky S. (2008). Rethinking rumination. Perspectives on Psychological Science.

[bib30] Papageorgiou C., Wells A. (1999). Process and meta-cognitive dimensions of depressive and anxious thoughts and relationships with emotional intensity. Clinical Psychology and Psychotherapy.

[bib31] Rush A.J., Gullion C.M., Basco M.R., Jarrett R.B., Trivedi M.H. (1996). The Inventory of depressive symptomatology (IDS): psychometric properties. Psychological Medicine.

[bib32] Satorra A., Bentler P.M. (2001). A scaled difference chi-square test statistic for moment structure analysis. Psychometrika.

[bib33] Schermelleh-Engel K., Moosbrugger H., Müller H. (2003). Evaluating the fit of structural equation models: test of significance and descriptive goodness-of-fit measures. Methods of Psychological Research Online.

[bib34] Segerstrom S.C., Stanton A.L., Alden L.E., Shortridge B.E. (2003). A multidimensional structure for repetitive thought: What’s on your mind, and how, and how much?. Journal of Personality and Social Psychology.

[bib35] Segerstrom S.C., Tsao J.C.I., Alden L.E., Craske M.G. (2000). Worry and rumination: repetitive thought as a concomitant and predictor of negative mood. Cognitive Therapy and Research.

[bib36] Siegle G.J., Moore P.M., Thase M.E. (2004). Rumination: one construct, many features in healthy individuals, depressed individuals, and individuals with lupus. Cognitive Therapy and Research.

[bib37] Spielberger C.D., Gorsuch R.L., Lushene R., Vagg P.R., Jacobs G.A. (1983). Manual for the State-trait anxiety Inventory.

[bib38] Stöber J. (1998). Reliability and validity of two widely-used worry questionnaires: self-report and self-peer convergence. Personality and Individual Differences.

[bib39] Trapnell P.D., Campbell J.D. (1999). Private self-consciousness and the five-factor model of personality: Distinguishing rumination from reflection. Journal of Personality and Social Psychology.

[bib40] Treynor W., Gonzalez R., Nolen-Hoeksema S. (2003). Rumination reconsidered: a psychometric analysis. Cognitive Therapy and Research.

[bib41] Vasey M.W., Borkovec T.D. (1992). A catastrophizing assessment of worrisome thoughts. Cognitive Therapy and Research.

[bib42] Watkins E. (2004). Appraisals and strategies associated with rumination and worry. Personality and Individual Differences.

[bib43] Watkins E., Moulds M., Mackintosh B. (2005). Comparisons between rumination and worry in a non-clinical population. Behaviour Research and Therapy.

[bib44] Watkins E.R. (2008). Constructive and unconstructive repetitive thought. Psychological Bulletin.

[bib45] Zetsche U., Ehring T., Ehlers A. (2009). The effects of rumination on mood and intrusive memories after exposure to traumatic material: an experimental study. Journal of Behavior Therapy and Experimental Psychiatry.

